# Cytotoxic response of ovarian cancer cell lines to IFN-**γ** is associated with sustained induction of IRF-1 and p21 mRNA

**DOI:** 10.1038/sj.bjc.6690491

**Published:** 1999-06

**Authors:** F Burke, P D Smith, M R Crompton, C Upton, F R Balkwill

**Affiliations:** Biological Therapies Laboratory and Electron Microscopy Laboratory, Imperial Cancer Research Fund, 44 Lincoln's Inn Fields, London, WC2A 3PX, UK; Institute of Cancer Research, Haddow Laboratories, Sutton, Surrey, SM2 5NG, UK

**Keywords:** interferon-γ, ovarian cancer, p21, stat1, IRF-1

## Abstract

Intereferon-γ (IFN-γ) has some anti-tumour activity in human ovarian cancer. This cytokine inhibited proliferation in three of four ovarian cancer cell lines in vitro. We then compared the action of IFN-γ in two cell lines, one sensitive and one resistant to its growth inhibitory effects. IFN-γ signalling appeared normal in both cell lines, with stat1 DNA binding activity detectable at 30 min. Continuous exposure to IFN-γ for 2–3 days was necessary for an irreversible effect on cell growth and apoptosis in cells sensitive to growth inhibition. During this time there was an increase in mRNA for the CKI p21, but no alterations in mRNA levels for other members of the CKI family. Maintenance of p21 mRNA required continuous mRNA synthesis. mRNA for the transcription factor IRF-1 was also induced in growth inhibited cells with similar kinetics to those observed for p21. Maximal induction of both p21 and IRF-1 mRNA was observed after 2–3 days IFN-γ exposure as the cells became committed to cell death. There was also a rapid increase in p21 and IRF-1 mRNA in cells resistant to the growth inhibitory effects of IFN-γ, but this increase was not maintained. Thus, continuous interaction with the IFN-γ receptor, together with a sustained induction of p21 and IRF-1, is associated with growth inhibitory and apoptotic effects of IFN-γ in ovarian cancer cells. © 1999 Cancer Research Campaign


*British Journal of Cancer* (1999) **80**(8), 1236–1244
© 1999 Cancer Research Campaign
Article no. bjoc.1999.0491

### Cytotoxic response of ovarian cancer cell lines to IFN-γ is associated with sustained induction of IRF-1 and p21 mRNA

**F Burke[1], PD Smith[2], MR Crompton[2], C Upton[3] and FR Balkwill[1]**

[1]Biological Therapies Laboratory and [2]Electron Microscopy Laboratory, Imperial Cancer Research Fund, 44 Lincoln's Inn Fields, London WC2A 3PX, UK; [3]Institute of Cancer Research, Haddow Laboratories, 15 Cotswold Road, Sutton, Surrey SM2 5NG, UK

**Summary** Intereferon-γ (IFN-γ) has some anti-tumour activity in human ovarian cancer. This cytokine inhibited proliferation in three of four ovarian cancer cell lines in vitro. We then compared the action of IFN-γ in two cell lines, one sensitive and one resistant to its growth inhibitory effects. IFN-γ signalling appeared normal in both cell lines, with stat1 DNA binding activity detectable at 30 min. Continuous exposure to IFN-γ for 2–3 days was necessary for an irreversible effect on cell growth and apoptosis in cells sensitive to growth inhibition. During this time there was an increase in mRNA for the CKI p21, but no alterations in mRNA levels for other members of the CKI family. Maintenance of p21 mRNA required continuous mRNA synthesis. mRNA for the transcription factor IRF-1 was also induced in growth inhibited cells with similar kinetics to those observed for p21. Maximal induction of both p21 and IRF-1 mRNA was observed after 2–3 days IFN-γ exposure as the cells became committed to cell death. There was also a rapid increase in p21 and IRF-1 mRNA in cells resistant to the growth inhibitory effects of IFN-γ, but this increase was not maintained. Thus, continuous interaction with the IFN-γ receptor, together with a sustained induction of p21 and IRF-1, is associated with growth inhibitory and apoptotic effects of IFN-γ in ovarian cancer cells.

**Keywords:** interferon-γ; ovarian cancer; p21; stat1; IRF-1

Interferon-γ (IFN-γ) is a potent immunomodulatory and anti-proliferative cytokine. The mechanisms(s) by which it inhibits tumour cell growth and induces apoptosis has yet to be fully elucidated. IFN-γ has shown some success in the treatment of ovarian cancer patients with minimal residual disease (Pujade-Lauraine et al, 1996). We previously demonstrated that human IFN-γ treatment of human ovarian cancer xenografts in vivo induces tumour cell growth arrest and apoptosis and increases mouse survival, although not all xenografts respond (Burke et al, 1997).

One potential mechanism by which IFN-γ inhibits cell growth is via increased expression of the cyclin kinase inhibitor (CKI) leading to a block in cell cycle progression and arrest of cell growth (Chin et al, 1996). In addition to IFN-γ, a number of other cytokines also display growth inhibitory properties, some of which have also been associated with CKI induction. For example, p21 has been implicated in growth inhibition in response to transforming growth factor β (TGF-β) in ovarian cancer cells (Elbendary et al, 1994); to tumour necrosis factor α (TNF-α) in a breast cancer cell line (Jeoung et al, 1995); to epidermal growth factor (EGF) in a squamous cell carcinoma cell line (Fan et al, 1995; Jakus and Yeudall, 1996); and to IFN-α in haematopoietic cell lines (Hobeika et al, 1997; Sangfelt et al, 1997). Another CKI, p27, has also been associated with IFN-γ-induced terminal growth arrest in mammary carcinoma cell lines (Harvat et al, 1997).

The intracellular signalling pathways which follow binding to IFN-γ-specific cell surface receptors have been well-characterized and involve the activation of members of the Janus kinase family (Jaks), Jak 1 and Jak 2 and stat1 (for signal transducer and activator of transcription (for review see Leaman et al, 1996). Data suggest that transcriptionally active stat1 is required for the anti-proliferative effects of IFN-γ (Bromberg et al, 1996; Buard et al, 1998). A mutant cell line displaying only 20–30% of wild-type transcriptional response did not undergo growth arrest in response to IFN-γ. Furthermore, activated stat1 proteins specifically recognized the conserved stat-responsive element in the promoter of the p21 gene and thus regulated the induction of p21 mRNA (Chin et al, 1996). In addition to stat1 activation in response to IFN-γ, the transcription factor IRF-1 also plays a role in the regulation of cell growth and apoptosis (reviewed by Taniguchi et al, 1997), and the induction of p21 in response to γ-irradiation is dependent on both IRF-1 and wild-type p53 (Tanaka et al, 1996).

In order to optimize the use of growth inhibitory/apoptotic cytokines such as IFN-γ in ovarian cancer, we set out to assess the following: the length of exposure to IFN-γ required for apoptosis of ovarian cancer cells; the signalling pathways that may correlate with cell growth inhibition and apoptosis; and the differences between IFN-γ-sensitive and -resistant cells. We report here that exposure to IFN-γ for at least 2 days was necessary for an anti-proliferative and apoptotic effect. Both sensitive and resistant cells contained proteins able to bind a stat1-responsive element. p21 and IRF-1 were also induced in both sensitive and resistant cells but this enhanced expression was only sustained in cells that were subsequently killed by IFN-γ.

*Received 5 October 1998*
*Revised 4 January 1999*
*Accepted 6 January 1999*

*Correspondence to:* F Burke

### METHODS

All reagents were provided by Sigma (Dorset, UK) unless otherwise stated.

**Table 1** Percentage of apoptotic cells as assessed by both 'Tunel' assay and morphometry. A minimum of ten high-power fields (100 × objective, 10 × eyepiece) were counted per cell line per time point and a mean taken

| Cell line | IFN-γ U ml$^{-1}$ | No. of cells counted | No. of apoptotic cells | Apoptotic cells (%) |
|---|---|---|---|---|
| PE01 | 0 | 465 | 23 | 4.9 |
| | 200 | 318 | 33 | 10.3 |
| | 5000 | 426 | 74 | 17.3 |
| SKOV-3 | 0 | 428 | 8 | 1.9 |
| | 200 | 343 | 11 | 3.2 |
| | 5000 | 469 | 15 | 3.2 |

Assessment of apoptosis using the 'Tunel' assay together with morphology. Numbers represent the mean number of apoptotic cells per ten high-power fields (× 1000) following 4 days treatment with IFN-γ.

### Tissue culture

All cell lines were grown in a humidified atmosphere at 37°C (5% carbon dioxide) under pyrogen-free conditions. Pasteur pipettes were triple baked and all other pipettes were plastic. Tissue culture medium and fetal calf serum (FSC; Life Technologies, Paisley, UK) were chosen for their low endotoxin content. Cells were grown in either RPMI-1640 or DMEM (SKOV-3) and 10% FCS. With the exception of SKOV-3 all cell lines were also grown in the presence of insulin (5 μg ml$^{-1}$). A total of 2 mM L-glutamine, 100 U ml$^{-1}$ penicillin and 100 μg ml$^{-1}$ of streptomycin were included in the tissue culture medium.

### Cell lines

PE01 was derived from ascites in a patient with poorly differentiated adenocarcinoma. PE014 was obtained prior to chemotherapy from a patient with well-differentiated serous adenocarcinoma (both obtained and described in Langdon et al, 1988). OVCAR-3 originated from ascites in a patient with poorly differentiated papillary adenocarcinoma (American Tissue Culture Collection (ATTC), Rockville, MD, USA). SKOV-3 originated from ascites in a patient with an ovarian adenocarcinoma and was also obtained from ATCC. Cell lines were treated with a range of doses (200, 1000, or 5000 U ml$^{-1}$) of recombinant human IFN-γ (rhIFN-γ). Viable cells were clearly identifiable and counted using a haemocytometer. PE01, PE014 and OVCAR-3 were all sensitive to the growth effects of IFN-γ, whilst SKOV-3 cells were resistant.

### Interferon rhIFN-γ (RU 42369) was provided by Roussel UCLAF, as a lyophilized preparation (20 MU per vial) and was more than 95% pure. It was diluted in water and stored in aliquots at – 70°C prior to use. Endotoxin levels were less than 0.24 UE per vial and the specific activity was $2 \times 10^7$ units per mg protein.

### Cell cycle analysis

Cells were seeded in 10-cm$^2$ dishes at concentrations that would ensure that the cells were semi-confluent at the time of harvest. At appropriate time points, cells were pulsed for 45 min with 10 mM bromodeoxyuridine (BrdU). The medium was subsequently removed, cells were washed, scraped into phosphate-buffered saline (PBS) and pelleted. The pellet was resuspended in 75% ethanol and stored at 4°C prior to analysis. Labelling for BrdU and DNA content were carried out following manufacturer's instructions provided with the fluorescein isothiocyanate (FITC)-conjugated anti-BrdU antibody (Becton Dickinson, UK). The cells were counter-stained with propidium iodide, and G1, S and G2 and were gated out as appropriate by an experienced FACS operator.

### Tunel assay

Cells were seeded in 10-cm$^2$ dishes as outlined above, and approximately $5 \times 10^5$ cells used to make cytospins. The cells were fixed in 4% paraformaldehyde and stored at room temperature prior to use. The method used was as described previously (Gavrieli et al, 1992).

### Electron microscopy

PE01 cells were incubated for 7 days at either 0, 200 or 5000 U ml$^{-1}$ of IFN-γ. The cells were trypsinized and resuspended in 2.5% glutaraldehyde in Sorensen's phosphate buffer and processed for electron microscopy using standard procedures. One μm toluidine blue stained sections were used to select representative areas for ultrastructural investigation and for cell counting.

### Northern analysis

Total cellular RNA was isolated using TRI REAGENT™ following manufacturer's instructions. Fifteen micrograms total cellular RNA was electrophoresed through a 1.4% agarose–formaldehyde denaturing gel and capillary blotted onto 'Biodyne A' membranes (Pall Ultrafine Filtration Corp, Glen Cove, NY, USA). cDNA was labelled with $^{32}$P-dCTP using a random priming kit (Stratagene, Cambridge, UK). Membranes were hybridized to the $^{32}$P-labelled inserts of human cDNA probes under standard conditions as outlined previously (Church and Gilbert, 1984). Membranes were subsequently washed to high-stringency and exposed to Kodak BioMax MS-1 film at –70°C with one intensifying screen.

### p21 mRNA synthesis

To assess the requirement for continued p21 mRNA synthesis, PE01 cells were treated with IFN-γ (5000 U ml$^{-1}$) for 24 h. Actinomycin D (10 μg ml$^{-1}$) was added to cells and RNA harvested 1, 3, 5 and 7 h later. Northern analysis was performed as outlined above.

*© 1999 Cancer Research Campaign* 

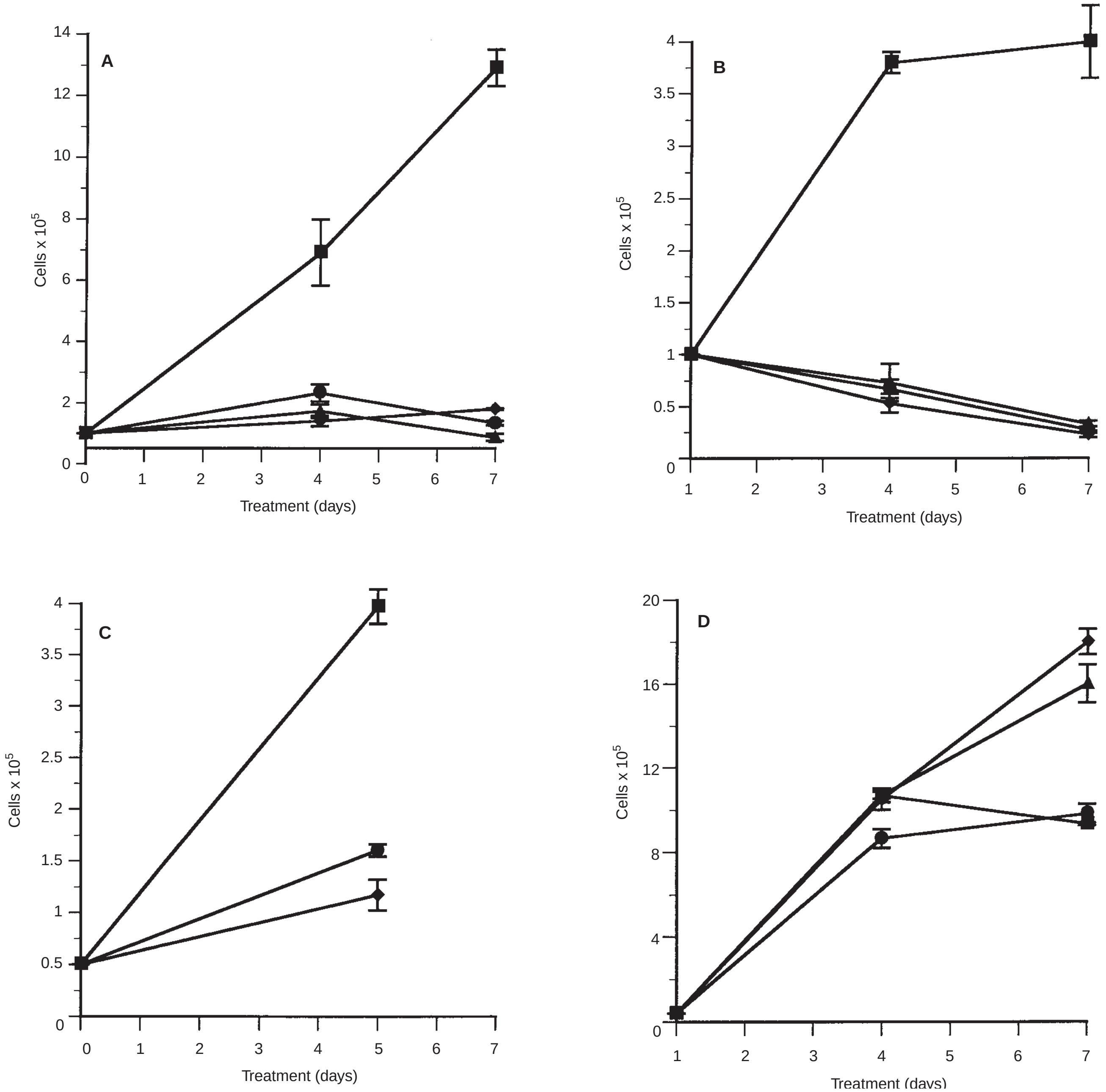

**Figure 1** Cell growth assays. Growth assays of PE01 (**A**), OVCAR-3 (**B**), PE014 (**C**) and SKOV-3 (**D**) cells grown in 10% FCS. Cells were seeded at $5 \times 10^4$ per well in 6-well plates. Each time point was carried out in triplicate. ■, Control; ●, IFN-γ 200 U $ml^{-1}$; ▲, IFN-γ 1000 U $ml^{-1}$; ◆, 5000 U $ml^{-1}$ IFN-γ

### cDNA probes

β-actin originated from Dr L Kedes (Stanford, CA, USA). The 0.7-kb *Eco*R1–*Hin*dIII fragment in Bluescript was used. The 486-bp *Nco*I cDNA fragment of p21 in pET21d-2 used for Northern analysis was kindly provided by Dr T Hunt (ICRF, Clare Hall Laboratories, South Mimms, UK). cDNAs for p15, 16, 18, 19, 27 were kindly provided by Dr G Peters (ICRF, PO Box 123, London, UK). All were in Bluescript (KS+ and fragments isolated with *Bam*HI–*Eco*RI (for p16, 18, 19, 27) or *Eco*RI–*Hin*dIII (p15). IRF-1 cDNA was provided by Dr Richard Pine (Public Health Research, New York, NY, USA). This was extracted as an *Eco*R1 insert from pBluescript SK.

### Cell extract preparations and EMSA

DNA-binding protein extracts were prepared as described previously (Andrews and Faller, 1991) with minor modifications. Briefly, $2 \times 10^6$ cells were washed in ice-cold PBS, pelleted and resuspended in lysis buffer (10 mM HEPES, pH 7.9, 10 mM potassium chloride, 1 mM dithiothreitol, 1 mM EDTA, 1 mM EGTA, 0.2% NP-40, 0.5 mM phenylmethylsulphonyl fluoride, 1 mM sodium vanadate, 1 mM β-glycerol phosphate, 20 mM sodium fluoride, 1 μg $ml^{-1}$ of aprotinin, leupeptin and pepstatin. Following their resuspension, the extracts were resuspended in buffer containing 25% glycerol and stored at –70°C prior to use. For EMSA, double stranded oligonucleotide for M67-SIE (GTGCATTTCCCGTAAATCTTGTCTACA) or a

*© 1999 Cancer Research Campaign*

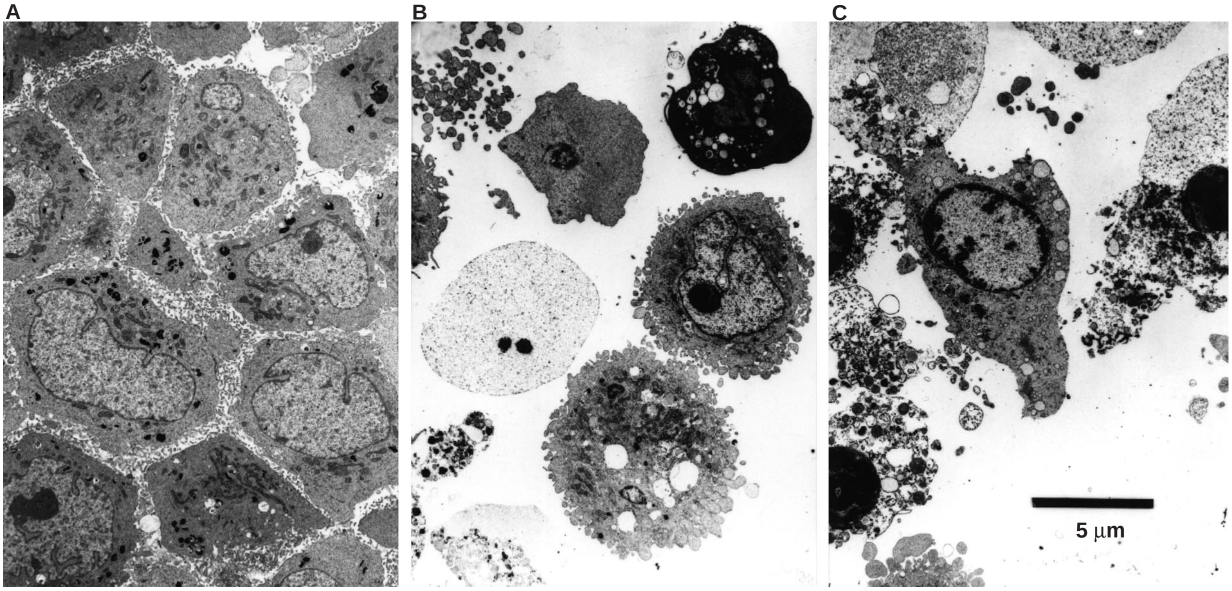

**Figure 2** Electron micrographs of PE01 cells cultured for 7 days. Cells were trypsinized prior to fixation. (**A**) Untreated, normal healthy tumour cells. (**B**) IFN-γ at 200 U ml$^{-1}$, culture contains normal and abnormal tumour cells, an apoptotic cell and cell debris. (**C**) IFN-γ at 5000 U ml$^{-1}$. Early and late apoptotic cells are evident, together with an apoptotic fragment and a necrotic cell

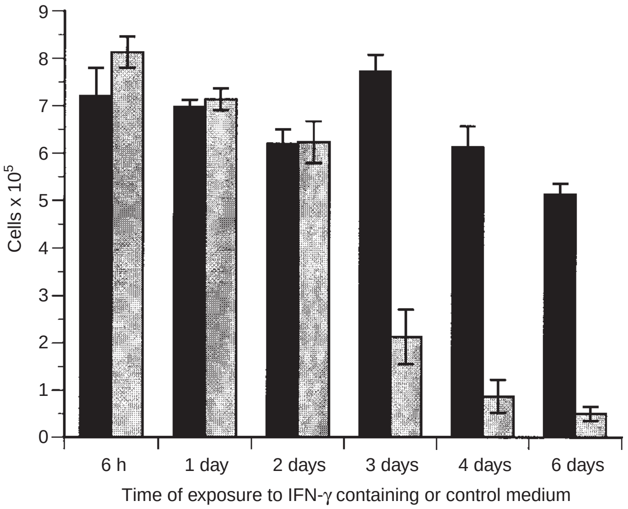

**Figure 3** Time of IFN-γ exposure to PE01 cells. PE01 cells were incubated with IFN-γ (5000 U ml$^{-1}$ for times between 6 h and 6 days, after which the cells were trypsinized, reseeded at 5 × 10$^4$ cells per well in 6-well plates and left for a further period of 6 days prior to counting. ■, Untreated cells; □, IFN-γ treated cells (5000 U ml$^{-1}$)

mutant SIE (GTGCATCCACCGTAAATCTTGTCTACA) was endlabelled with [γ-$^{32}$P] adenosine 5′-triphosphate according to standard procedures. Binding reactions were performed in a total volume of 20 μl in 40 mM HEPES, pH 7.9, 2 mM magnesium chloride, 8% Ficoll, 2 mM dithiothreitol, 100 mM sodium chloride, poly(deoxyinosine–deoycytidine) (Pharmacia, 50 μg ml$^{-1}$). Extracts were incubated with labelled oligonucleotides at room temperature for 20 min. Duplicate samples were also set up containing 50 times excess of cold oligonucleotide as a competitor. Complexes were separated on 4% acrylamide gels in 0.25 times Tris–borate EDTA at 4°C, dried down and detected by autoradiography.

### Protein estimations and PAGE analysis

Protein estimations were carried out using the Bio-Rad protein assay (Life Science Research Products, Herts, UK), and samples were boiled for 2 min and stored on ice prior to loading. A 10% denaturing gel was made up for detection of stat1 protein. The lysates were run at 120 V for 1.5 h on a AE-6400 Dual Mini slab kit (Atto) using 1 × running buffer (25 mM Tris; 192 mM glycine; 0.1% sodium dodecyl sulphate). Rainbow markers (Amersham) were used to determine product size.

### Western blotting

Protein was transferred to nitrocellulose (Hybond-ECL, Amersham, UK) which had been pre-equilibrated in transfer buffer (25 mM Tris, 192 mm glycine, 20% methanol, pH 8.3) according to the method of Towbin et al, 1979) using a Trans-Blot electrophoretic transfer cell (Biorad) for 2–3 h at 300 mA. Blots were incubated in blocking reagent (10% non-fat milk powder) overnight. Proteins were detected using Amersham ECL™. For Stat1, the cytosolic and nuclei components obtained for the EMSA assay were used (10 μg per track) and monoclonal antibody used at 1 μg ml$^{-1}$ (Santa Cruz, Autogen Bioclear, Wiltshire, UK).

### Statistics

The unpaired Student's *t*-test was used for statistical analysis.

### RESULTS

### Direct cell counting as a measure of cell growth

Four human ovarian cancer cell lines were grown under optimal conditions in 10% FCS and assessed for their responsiveness to a range of concentrations of IFN-γ. In three of these lines. (PE01, PE014 and OVCAR-3), IFN-γ inhibited cell growth as shown in

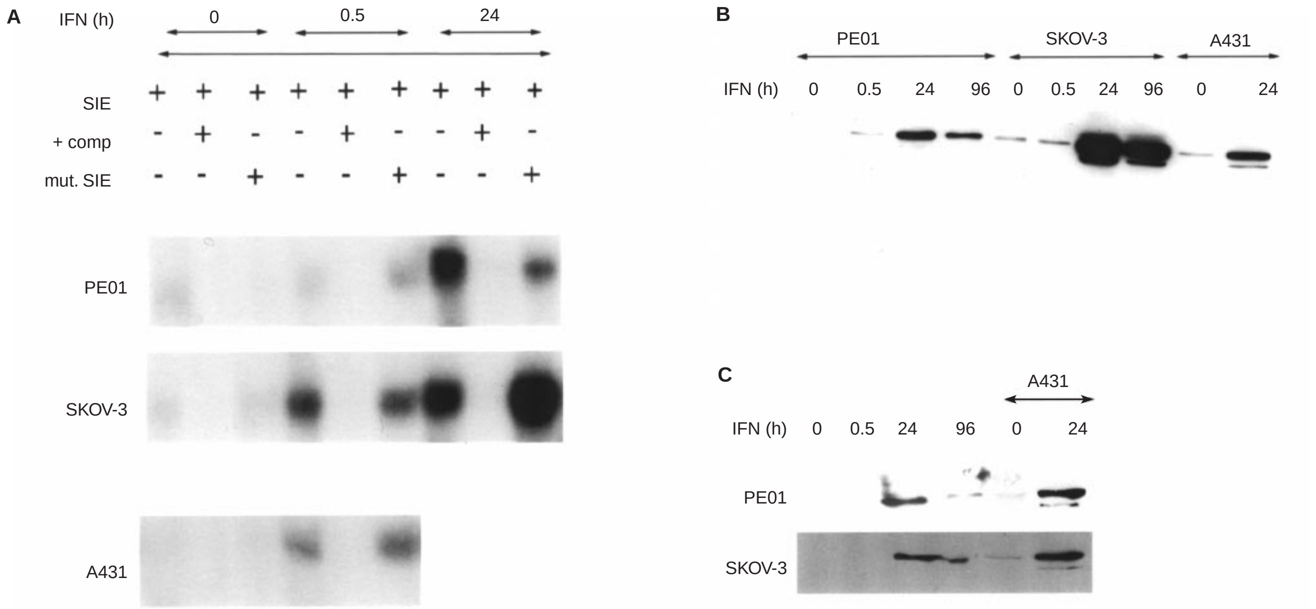

**Figure 4** Stat1 binding activity and protein in IFN-γ-sensitive and resistant cells. (**A**) EMSA: Stat complexes were detected using M67-SIE, together with excess cold competitor and mutant SIE. IFN-γ-treated A431 cells were used as a positive control for stat1 activation. (**B**) Stat-1 protein expression in SKOV-3 and PE01 cells following treatment with IFN-γ (200 U $ml^{-1}$). Ten micrograms of cellular cytosolic component (**B**) or nuclei component (**C**) was loaded per track. Western blotting analysis carried out as described in Methods

Figure 1. After 4–5 days of IFN-γ treatment at 200 U $ml^{-1}$, cell counts in three cell lines were 29–40% of untreated cultures and this was further reduced to 10–14% of untreated values by 7 days. The effects of IFN-γ were not dependent on optimal growth conditions. IFN-γ was also growth inhibitory for two cell lines (PE01 and OVCAR-3) that were cultured in the absence of serum (data not shown).

The IFN-γ sensitive PE01 cells were selected for further analysis. The minimum concentration of IFN-γ required to achieve growth inhibition in this cell line was 100 U $ml^{-1}$ (data not shown), and at 200, 1000 and 5000 U $ml^{-1}$ significant growth inhibition was seen after 4 and 7 days exposure ($P < 0.0001$ at 7 days for untreated vs all three doses). In contrast, SKOV-3 cells were resistant to the growth inhibitory actions of IFN-γ at 200, 1000 and 5000 U $ml^{-1}$.

As a further measure of growth inhibition in sensitive cells, we used the thymidine analogue BrdU to assess the number of PE01 cells in S phase of the cell cycle. The time course of growth inhibition followed the same pattern as that described above. By 4 days, only 10% of IFN-γ-treated cells were synthesizing DNA compared to 24% untreated cells.

### IFN-γ and apoptosis

The 'Tunel' assay was used together with an assessment of morphology to measure cell death in treated cultures. These results are shown in Table 1 and represent the mean number of apoptotic cells per ten high power fields (× 1000) following 4 days of treatment with IFN-γ. There was an increase in apoptosis after IFN-γ treatment of PE01 cells compared to untreated cells. The percentage of cells that were apoptotic increased from 4.9% in untreated to 17.3% following 4 days treatment of 5000 U $ml^{-1}$ of IFN-γ. In contrast, the number of apoptotic cells did not significantly change throughout treatment in SKOV-3.

Apoptosis was further investigated using electron microscopy following treatment of PE01 with IFN-γ for 7 days. Cells were counted and assigned to a category on the basis of their general appearance, with particular attention to the nucleus. The control culture contained normal, healthy tumours cells (Figure 2A). The culture that had been treated with IFN-γ at 200 U $ml^{-1}$ had a high proportion of healthy normal cells, some abnormal and a considerably increased number of apoptotic cells (Figure 2B). Approximately half those cells treated with IFN-γ at 5000 U $ml^{-1}$ were dead, most of them being apoptotic (Figure 2C). The total number of apoptotic cells increased from 0.3% in untreated cells to 4.2% with IFN-γ (200 U $ml^{-1}$), and 30.7% with 5000 U $ml^{-1}$ of IFN-γ for 7 days.

### Length of exposure to IFN-γ

In order to assess how long IFN-γ had to interact with the cells to obtain maximal growth inhibitory effects, PE01 cells were incubated with IFN-γ for periods of 6 h to 6 days. Simply washing the cells after these time points was not sufficient to remove IFN-γ bound to its receptor. Therefore, at appropriate times, IFN-γ-treated cells were trypsinized, washed and re-seeded ($5 \times 10^4$ viable cells per well in 6-well plates) in culture medium without IFN-γ. After a further 6 days culture, cells were counted. These results are shown in Figure 3. If cells were exposed to IFN-γ for between 6 and 48 h prior to trypsinization, they subsequently grew to reach similar numbers as previously untreated cells. If cells were treated with IFN-γ for longer than 48 h prior to trypsinization, they were not able to proliferate to the same extent when replated in normal culture medium. Cell recovery was reduced by 72%, 86% and 90% as compared to untreated cells for 3 ($P$ = 0.0003), 4 ($P$ = 0.0007) and 6 ($P$ = 0.0001) days prior to IFN-γ exposure respectively.

*© 1999 Cancer Research Campaign*

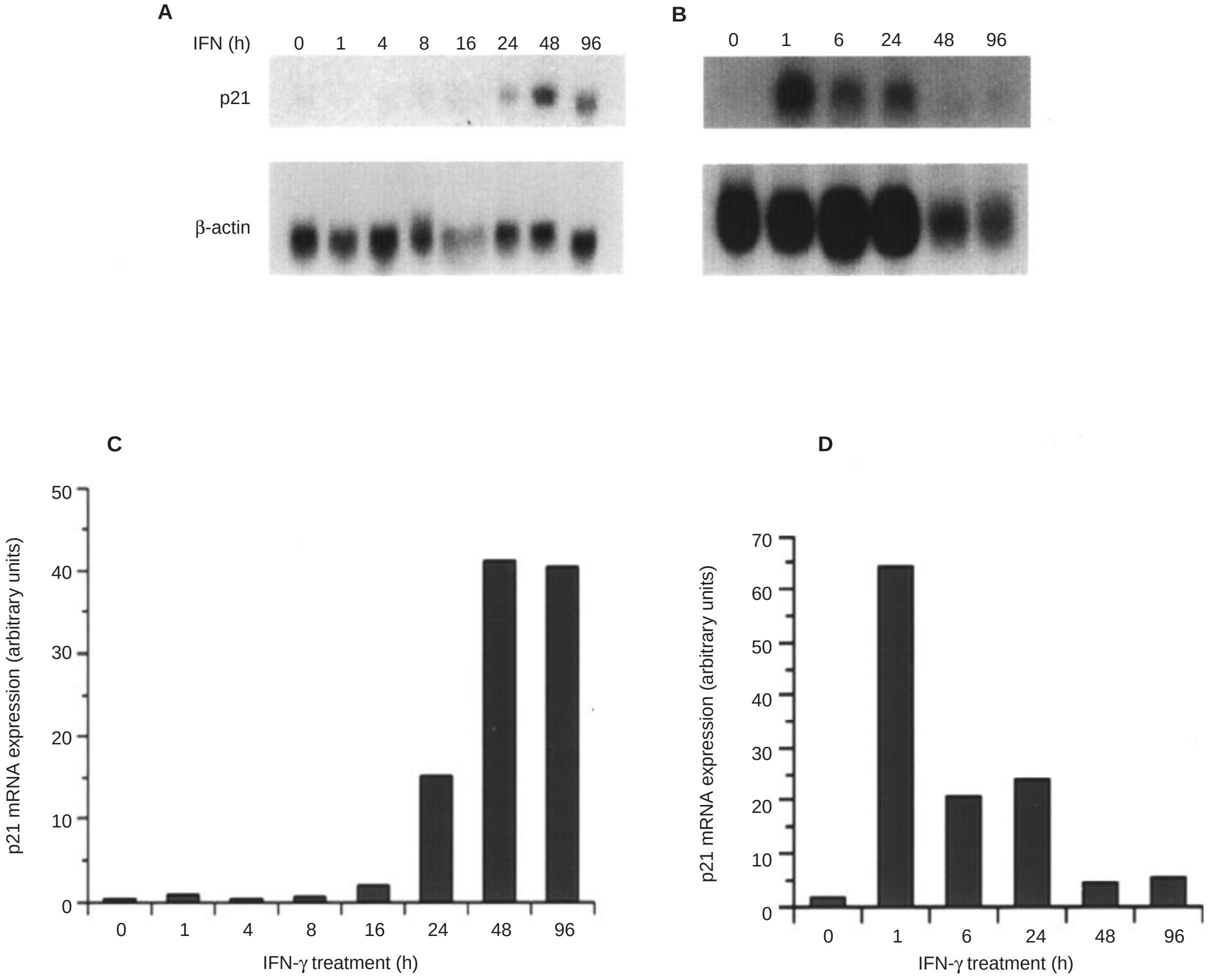

**Figure 5** p21 mRNA expression of IFN-γ-treated sensitive and resistant cells. (**A**) PE01 cells. The expression of mRNA for CKI p21 following IFN-γ (5000 U $ml^{-1}$) treatment. The blot was reprobed with β-actin as a loading control. Fifteen micrograms of total RNA were loaded per lane. (**C**) Integrated density of p21 mRNA in PE01 cells after correcting for β-actin. (**B**) SKOV-3 cells. The expression of mRNA for CKI p21, following IFN-γ (5000 U $ml^{-1}$) treatment. The blot was reprobed with β-actin as a loading control. Fifteen micrograms of total RNA were loaded per lane. (**D**) Integrated density of p21 mRNA in SKOV-3 cells after correcting for β-actin

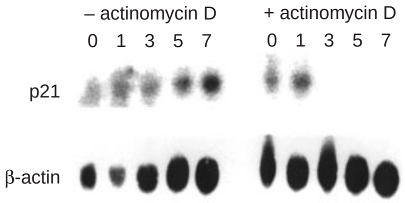

**Figure 6** p21 mRNA stability of IFN-γ-treated PE01 cells. PE01 cells were treated with IFN-γ (5000 U $ml^{-1}$) for 24 h. Cells were subsequently treated with actinomycin D (10 μg $ml^{-1}$) for 1, 3, 5, 7 h. Control cells were treated with IFN-γ only. The maintenance of p21 mRNA required continuing RNA synthesis

To further assess the molecular events that occur following IFN-γ treatment of ovarian cell lines, we studied PE01 and SKOV-3 cells that were sensitive and resistant to the growth inhibitory and apoptotic effects of IFN-γ respectively.

### Stat-1 and the anti-proliferative effect of IFN-γ

The ability of a cell to induce stat1 may be a requirement for an anti-proliferative response to IFN-γ. As shown in Figure 4A, PE01 cells had *cis*-inducible element-binding ability. This was subsequently confirmed by Western blotting. Figure 4B and 4C shows the detection of stat1 in the cytosolic and nuclear compartments of the cells. Figure 4A shows that SKOV-3 cells also had stat1-binding activity. Indeed, there was clearly more protein for stat1 in both cytoplasmic and nuclear compartments in the SKOV-3 cells compared to PE01.

### mRNA expression of CKIs following IFN-γ treatment in ovarian cancer cell lines

Northern analysis was used to assess changes in CKI mRNA expression during IFN-γ treatment of PE01 and SKOV-3 cells over a period of 96 h. Members of both the INK family (p16, 15, 18, 19) and the p21 (p21, p27) family of CKIs were analysed at the mRNA level. However, only p21 mRNA expression differed significantly throughout treatment in both cell lines. In PE01 cells,

© 1999 Cancer Research Campaign 

p21 mRNA was undetectable or expressed at low levels in IFN-γ-treated cells until 16 h. p21 mRNA increased at 24 h and this increase was sustained at 48 and 96 h of IFN-γ treatment. Densitometry showed an approximately 15-fold increase in mRNA expression at 24 h, which further increased to 40-fold at 48 and 96 h (Figure 5C, arbitrary units relative to β-actin). We also assessed the p21 mRNA level in another sensitive cell line OVCAR-3. The same pattern of mRNA expression was observed with maximal effects at 48 h (data not shown).

In the SKOV-3 cells, which were not growth inhibited by IFN-γ, p21 mRNA was also induced but with different kinetics to those observed with PE01 cells (Figure 5B). There was a rapid 60-fold induction of p21 mRNA after just 1 h of treatment with IFN-γ, which gradually decreased thereafter (Figure 5D), arbitrary units relative to β-actin). At a time when the p21 mRNA levels were maximal in the IFN-γ-sensitive PE01 cells that were irreversibly growth arrested, the mRNA expression of p21 in SKOV-3 cells was not significantly different from untreated cells.

### p21 mRNA synthesis

We used actinomycin D treatment to see whether the gradual accumulation in p21 mRNA was associated with continuous mRNA synthesis. PE01 cells were treated with IFN-γ for 24 h. At this time, actinomycin D was added to cells and RNA was harvested during a 7-h time period. Cells treated with IFN-γ alone were used as controls. p21 mRNA was detectable in IFN-γ- and actinomycin D-treated cells up to 3 h, after which it was no longer detectable. As expected, p21 was detectable in all time points analysed where cells had been treated with IFN-γ alone (Figure 6). This experiment clearly demonstrates that p21 mRNA synthesis must be continuous.

### IRF-1 mRNA expression of cells in response to IFN-γ

IRF-1 has been associated with IFN-γ-induced cell growth inhibition and apoptosis (Taniguchi et al, 1997) and the induction of p21 in response to γ-irradiation is dependent on both IRF-1 and p53 (Tanaka et al, 1996). We assessed IRF-1 mRNA expression in IFN-γ-treated PE01 and SKOV-3 cells. In the PE01 cells there was a gradual increase in mRNA which peaked at 48 h (Figure 7A, B), arbitrary units relative to β-actin) and the induction was greater than in untreated cells. The kinetics of expression of IRF-1 mRNA in another IFN-γ-sensitive cell line, OVCAR-3, resembled those of PE01 cells (data not shown).

The treatment of SKOV-3 cells with IFN-γ resulted in a rapid increase of IRF-1 mRNA at 1 and 6 h. However, this induction was not sustained and the pattern of expression at later time points was similar to untreated cells (Figure 7C, D, arbitrary units relative to β-actin).

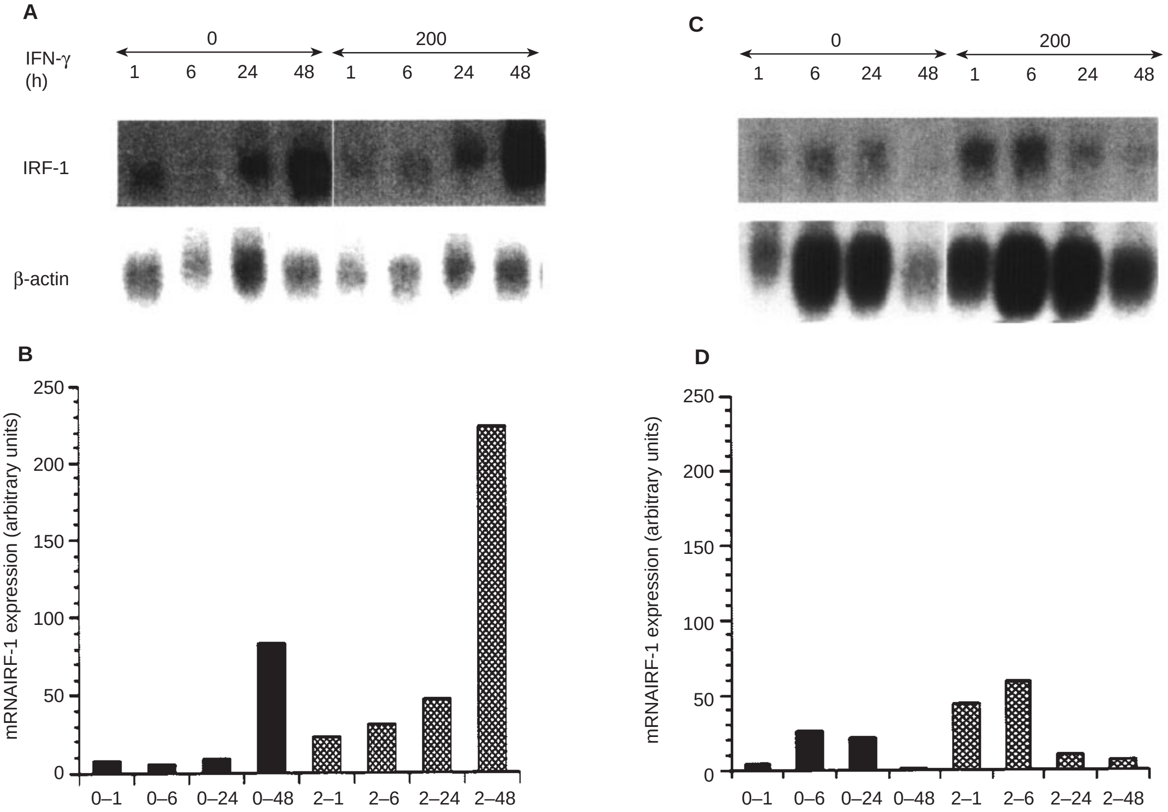

**Figure 7** IRF-1 mRNA expression of IFN-γ-treated sensitive and resistant cells. (**A**) PE01 cells. IRF-1 mRNA expression in untreated and IFN-γ treated (200 U $ml^{-1}$). The blot was reprobed with β-actin as a loading control. Fifteen micrograms of total RNA were loaded per lane. (**B**) Integrated density of IRF-1mRNA in PE01 after correcting for β-actin. (**C**) SKOV-3 cells. IRF-1 mRNA expression of untreated and IFN-γ-treated (200 U $ml^{-1}$) cells. The blot was reprobed with β-actin as a loading control. Fifteen micrograms of total RNA were loaded per lane. (**D**) Integrated density of IRF-1 mRNA in SKOV-3 cells after correcting for β-actin

 *© 1999 Cancer Research Campaign*

### DISCUSSION

IFN-γ displays growth inhibitory activities in a number of normal and malignant cell lines and this cytokine may be of therapeutic benefit to some patients with ovarian cancer (Pujade-Lauraine et al, 1996). In this study we have shown that IFN-γ is capable of inducing both growth inhibition and apoptosis in the ovarian cancer cell lines PE01, PE014 and OVCAR-3. In contrast, SKOV-3 cells continue to proliferate in response to IFN-γ.

The growth inhibitory effects of IFN-γ in PE01 cells are not immediate. It takes 3 days of continual IFN-γ exposure for any reduction in cell number and for an irreversible effect on cell survival. Exposing the PE01 cells to IFN-γ for a shorter period of time had no effect on their subsequent ability to grow over a period of 6 days. The importance of exposure time was highlighted in an earlier study where we reported that the treatment of human ovarian cancer xenografts with IFN-γ for at least 14 days in vivo was more effective than giving higher doses for shorter periods of time (Burke et al, 1997).

Since transcriptionally active stat1 is required for the anti-proliferative effect of IFN-γ (Bromberg et al, 1996), we examined the ability of resistant cells to recruit stat1 to the nucleus and assessed their DNA binding activity. Nuclear components from SKOV-3 cells demonstrated a stronger SIE-binding ability than those from PE01 cells, and Western blotting confirmed that there was a higher level of immunoreactive stat1. A recent paper has been published describing the jak/stat signalling pathway in mesothelioma cell lines with differing responses to IFN-γ (Buard et al, 1998). IFN-γ phosphorylated jak2 and stat1, and induced the transcription factor IRF-1 in three cell lines that were strongly growth inhibited. Activation of the jak/stat pathway was weak or absent in two further cell lines that were insensitive to IFN-γ-mediated growth inhibition. However, in two further cell lines, IFN-γ jak/stat signalling appeared normal, but the cells were only weakly responsive to IFN-γ. Thus, it would seem that cancer cell lines accumulate signalling defects at various points in, and downstream from, the jak/stat pathway. The data described here would support this conclusion.

One candidate gene downstream from the jak/stat pathway is the CKI p21 (Chin et al, 1996). We looked for the regulation of this CKI following IFN-γ treatment of PE01 and SKOV-3 cells by Northern analysis. Levels of p21 mRNA changed significantly during IFN-γ treatment of PE01 cells. There was a sustained induction of the mRNA between 24 and 96 h, which correlates well with the cell growth assay data where we found that sustained treatment was important. Western blotting for p21 showed that the protein was also undetectable until 24 h (unpublished data).

mRNA for p21 was also detectable by Northern analysis in the cell line SKOV-3. There was a rapid increase in p21 mRNA expression after only 1 h, which was not maintained. At a time when PE01 cells were expressing high levels of p21 and undergoing irreversible cell growth arrest, the p21 mRNA expression in SKOV-3 cells had returned to baseline levels. We were unable to detect any changes in either PE01 or SKOV-3 cells in response to IFN-γ in five other CKIs investigated, although p27 has been associated with mediating IFN-γ-induced terminal growth arrest in mammary carcinoma cell lines (Harvat et al, 1997).

The kinetics of p21 mRNA expression following IFN-γ exposure to PE01 cells were different from that following TGF-β1 treatment of the same cells, but again there was a correlation between p21 kinetics and growth inhibition (data not shown). PE01 cells were growth inhibited by TGF-β1, and p21 mRNA was induced after only 1 h IFN-γ treatment, but the increase was then sustained. The kinetics of induction confirm previously published data on other ovarian cell lines (Elbendary et al, 1994). TGF-β treatment of SKOV-3 cells did not significantly affect their growth compared to untreated cells ($P = 0.09$) and there were no changes in p21 mRNA throughout the treatment period.

Using actinomycin D we showed that the maintenance of the increased p21 mRNA required continous mRNA synthesis. Since we were unable to detect p21 mRNA in cells pretreated with IFN-γ after 3 h of actinomycin D treatment, we performed transient transfection assays to directly assess p21 gene transcription in response to IFN-γ. The WWP-luc plasmid was provided by Dr Bert Vogelstein (John Hopkins University, USA) and carries the p21 promoter sequence fused to a promoterless luciferase gene. There was little difference in p21 promoter gene transcription in treated and untreated cells. It is likely, therefore, that the response to IFN-γ requires regulatory regions of the gene that are not in this reporter construct. Two of the three known SIE sites 5′ of the gene are outside the fragment in the construct. As a positive control for this experiment, we co-transfected PE01 cells with a p21 promoter construct together with wild-type p53 and demonstrated that the p53-dependent response was operational in PE01 cells when transfected with this construct (data not shown).

Finally, we assessed IRF-1 mRNA changes in PE01 and SKOV-3 cells in response to IFN-γ. IRF-1 mRNA gradually increased in IFN-γ treated PE01 cells relative to untreated cells, at a time when these cells become irreversibly growth arrested. This gradual increase in expression presented a very similar pattern to that observed with p21 mRNA. IRF-1 mRNA expression in SKOV-3 cells in response to IFN-γ also resembled that for p21. There was an initial induction which then slowly declined. Thus SKOV-3 cells were able to induce either p21 or IRF-1 in response to IFN-γ. However, there seemed be a defect in their ability to sustain the induction of these genes.

Our data suggest that there may be a critical time window between 48 and 72 h when PE01 cells become committed to a pathway leading to irreversible growth arrest and subsequent apoptosis when exposed to IFN-γ. A number of papers have been published recently suggesting target genes responsible for the anti-proliferative effects of IFN-γ. The data from these papers, together with the data we present here, suggest that different cell lines have signalling defects at different levels. For example, some cells may have defects in their ability to induce jaks or stats, whilst others have defects in being able to activate IRF-1. Other cells may have the ability to induce all of these, but are not able to sustain this induction. The SOCS (suppressor of cytokine signalling) proteins are a family of negative regulators of cytokine signal transduction (for review see Nicholson and Hilton, 1998). They are induced by stats and inhibit cytokine signal transduction via a negative feedback loop. One member of this family, JAB, is strongly induced by IFN-γ and its overexpression may be involved in IFN-γ resistance in some cells (Sakamoto et al, 1998). It is possible that our IFN-γ-insensitive cell line may overexpress one or more of these proteins.

Preliminary data from this laboratory suggest that tumour cells from freshly isolated ovarian cancer as cites behave in a similar way to the cell lines studied here (L Wall, unpublished data). Whilst tumour cells from most ascitic samples undergo apoptosis in response to IFN-γ, a minority are resistant. The molecular analysis described here may help in determining markers of

© 1999 Cancer Research Campaign 

response to IFN-γ and in the identification of patients most likely to benefit from IFN-γ therapy.

### ACKNOWLEDGEMENTS

We would like to thank Derek Davies for FACS analysis, and Drs Gordon Peters and Tim Hunt for useful comments and discussion.

### REFERENCES

Andrews NC and Faller DV (1991) A rapid micropreparation technique for extraction of DNA-binding proteins from limiting numbers of mammalian cells. *Nucleic Acids Res* **19**: 2499

Bromberg JF, Horvath CM, Wen ZL, Schreiber RD and Darnell JE (1996) Transcriptionally active stat1 is required for the antiproliferative effects of both interferon-alpha and interferon-gamma. *Proc Natl Acad Sci USA* **93**: 7673–7678

Buard A, Vivo C, Monnet I, Boutin C, Pilatte Y and Jaurand M (1998) Human malignant mesothelioma cell growth: activation of Janus kinase 2 and signal transducer and activator of transcription 1α for inhibition by interferon-γ. *Cancer Res* **58**: 840–847

Burke F, East N, Upton C, Patel K and Balkwill FR (1997) Interferon gamma induces cell cycle arrest and apoptosis in a model of ovarian cancer: enhancement of effect by batimastat. *Eur J Cancer* **33**: 1114–1121

Chin YE, Kitagawa M, Su WCS, You ZH, Iwamoto Y and Fu XY (1996) Cell-growth arrest and induction of cyclin-dependent kinase inhibitor p21 (waf1/cip1) mediated by stat1. *Science* **272**: 719–722

Church GM and Gilbert W (1984) Genomic sequencing. *Proc Natl Acad Sci USA* **81**: 1991–1995

Elbendary A, Berchuck A, Davis P, Havrilesky L, Bast RC, Jr, Iglehart JD and Marks, JR (1994) Transforming growth factor beta 1 can induce CIP1/WAF1 expression independent of the p53 pathway in ovarian cancer cells. *Cell Growth Differ* **5**: 1301–1307

Fan Z, Lu Y, Wu XP, Deblasio A, Koff A and Mendelsohn J (1995) Prolonged induction of p21 (cip1/waf1)/cdk2/PCNA complex by epidermal growth-factor receptor activation mediates ligand-induced A431 cell-growth inhibition. *J Cell Biol* **131**: 235–242

Gavrieli Y, Sherman Y and Ben Sasson SA (1992) Identification of programmed cell death in situ via specific labeling of nuclear DNA fragmentation. *J Cell Biol* **119**: 493–501

Harvat BL, Seth P and Jetten AM (1997) The role of p27 (kip1) in γ-interferon-mediated growth arrest of mammary epithelial cells and related defects in mammary-carcinoma cells. *Oncogene* **14**: 2111–2122

Hobeika AC, Subramaniam PS and Johnson HM (1997) IFN-α induces the expression of the cyclin-dependent kinase inhibitor p21 in human prostate-cancer cells. *Oncogene* **14**: 1165–1170

Jakus J and Yeudall WA (1996) Growth-inhibitory concentrations of EGF induce p21 (waf1/cip1) and alter cell-cycle control in squamous carcinoma-cells. *Oncogene* **12**: 2369–2376

Jeoung DI, Tang B and Sonenberg M (1995) Effects of tumor necrosis factor-alpha on antimitogenicity and cell cycle-related proteins in MCF-7 cells. *J Biol Chem* **270**: 18367–18373

Langdon SP, Lawrie SS, FG, H, Hawkes MM, McDonald MM, Hayward A, Ip S, Hilgers J, Leonard RCF and Smyth JF (1988) Characterisation and properties of nine human ovarian adenocarcinoma cell lines. *Cancer Res* **48**: 6166–6172

Leaman DW, Leung S, Li X and Stark GR (1996) Regulation of STAT-dependent pathways by growth factors and cytokines. *Faseb J* **10**: 1578–1588

Nicholson SE and Hilton DJ (1998) The SOCS proteins: a new family of negative regulators of signal transduction. *J Leukoc Biol* **63**: 665–668

Pujade-Lauraine E, Guastalla J-P, Colombo N, Devillier P, Franciois E, Fumoleau P, Monnier A, Nooy M, Mignot L, Bugat R, Marques C, Mousseau, M, Netter G, Maloisel F, Larbaoui S and Brandely M (1996) Intraperitoneal recombinant interferon gamma in ovarian cancer patients with residual disease at second-look laparotomy. *J Clin Oncol* **14**: 343–350

Sakamoto H, Yasukawa H, Masuhara M, Tanimura S, Sasaki A, Yuge K, Ohtsubo M, Ohtsuka A, Fujita T, Ohta T, Furukara Y, Iwase S, Yamada H and Yoshimura A (1998) A Janus kinase inhibitor, JAB, is an interferon-gamma-inducible gene and confers resistance to interferons. *Blood* **92**: 1668–1676

Sangfelt O, Erickson S, Einhorn S and Grander D (1997) Induction of cip/kip and INK 4 cyclin-dependent kinase inhibitors by interferon-α in hematopoietic-cell lines. *Oncogene* **14**: 415–423

Tanaka N, Ishihara M, Lamphier MS, Nozawa H, Matsuyama T, Mak TW, Aizawa S, Tokino T, Oren M and Taniguchi T (1996) Cooperation of the tumour suppressors IRF-1 and p53 in response to DNA damage. *Nature* **382**: 816–818

Taniguchi T, Lamphier MS and Tanaka N (1997) IRF-1: the transcription factor linking the interferon response and oncogenesis. *Biochim Biophys Acta* **1333**: M9–17

Towbin H, Staehelin T and Gordon J (1979) Electrophoretic transfer of proteins from polyacrylamide gels to nitrocellulose sheets: procedure and some applications. *Proc Natl Acad Sci USA* **76**: 4350–4354

 *© 1999 Cancer Research Campaign*
